# Limitations of CT-based planning in anterior pelvic ring fixation: a study of fluoroscopy-guided pubic ramus screws

**DOI:** 10.1007/s00590-026-04817-8

**Published:** 2026-06-19

**Authors:** Guillaume David, Thomas Grimaud, Clément Marc, Vincent Steiger, Louis Rony

**Affiliations:** 1 Centre Hospitalier Universitaire d’Angers, Angers, France; 2 Centre Hospitalier Universitaire de Bordeaux, Bordeaux, France

**Keywords:** Pubic ramus fracture, Anterior pelvic ring, Percutaneous screw fixation, Computed tomography planning, Surgical navigation

## Abstract

**Purpose:**

Current CT-based planning for pubic ramus screw fixation uses rectilinear geometric models based on the outer thread diameter to define intraosseous corridors. This method may underestimate feasibility by failing to incorporate implant geometry and non-rectilinear screw trajectories achievable under fluoroscopic guidance. This study aimed to determine the proportion of fluoroscopically inserted pubic ramus screws that would have been classified as infeasible using standard CT-based rectilinear planning and to explore mechanisms for this discrepancy.

**Methods:**

We conducted a retrospective cohort study at a Level I trauma center including all patients who underwent fluoroscopy-guided percutaneous pubic ramus screw fixation from January 2022 to December 2025 with complete pre- and postoperative pelvic CT imaging. The primary outcome was the proportion of screws judged infeasible under standard CT-based rectilinear planning using outer thread diameter. Secondary outcomes were interobserver reliability for feasibility assessment, postoperative screw positioning accuracy, complications, and the impact of core-diameter-based planning on feasibility classification.

**Results:**

Thirty-three patients (42 screws) were analyzed. Standard CT-based rectilinear planning classified 15 screws (36%) as infeasible by consensus of two observers. Postoperative CT demonstrated accurate positioning of all screws (100%) with no cortical breach, intra-articular penetration, neurovascular injury, or fixation-related revision. Core-diameter-based planning reclassified 14 of the 15 screws as feasible. In one case, three-dimensional postoperative reconstruction revealed a markedly non-rectilinear trajectory not approximable by any straight cylindrical model.

**Conclusion:**

Standard CT-based rectilinear planning underestimates the feasibility of fluoroscopically guided pubic ramus screw fixation. Core-diameter-based planning improves prediction but may still fail with pronounced trajectory curvature, highlighting limitations of rectilinear models for preoperative planning and navigation systems.

## Introduction

Percutaneous screw fixation of superior pubic ramus fractures is a widely accepted technique for stabilizing anterior pelvic ring injuries while limiting surgical morbidity [1–3]. Despite its advantages, accurate screw placement remains technically demanding because the intramedullary pathway of the pubic ramus is narrow, curved, and highly variable [4–8]. To assess feasibility and minimize the risk of cortical breach or intra-articular penetration, preoperative CT-based planning is commonly used to identify an osseous corridor suitable for screw insertion [9–11].

Most CT-based planning and navigation systems rely on rectilinear geometric assumptions, defining feasibility by the presence of a straight cylindrical corridor capable of accommodating a screw of predefined diameter. Using this approach, several CT simulation studies have reported limited feasibility rates. Lee et al. [9]. demonstrated that only 35% of patients could accommodate a 6.5 mm screw along a straight corridor and only 21.5% a 7.3 mm screw; notably, 12.9% of women were deemed unable to accept even a 3.5 mm screw. These findings suggest that strict rectilinear CT-based criteria may exclude a substantial proportion of patients from percutaneous anterior pelvic ring fixation.

Several methodological and biomechanical factors may contribute to this apparent limitation. First, feasibility is typically assessed using the outer thread diameter of the implant, although the risk of cortical violation is primarily determined by the screw core [9]. For commonly used 6.5 mm and 7.3 mm cannulated screws, the core diameter remains approximately 5 mm, while the increase in nominal diameter mainly reflects larger thread geometry. In narrow segments of the pubic ramus, particularly near the acetabular joint, planning based on thread diameter may therefore be overly restrictive [12]. In this context, the use of partially threaded screws allows the most constrained portion of the corridor to be traversed by the core alone, while threads engage wider cancellous regions, a strategy that is not captured by conventional CT planning models.

Second, rectilinear CT planning does not account for the intraoperative behavior of flexible guidewires. During fluoroscopy-guided cannulated screws insertion, guidewires may progressively adapt their trajectory within the intramedullary canal, undergoing controlled elastic deformation when encountering areas of denser cortical bone. The definitive screw subsequently follows this guidewire-defined path. Such non-rectilinear trajectories, potentially accentuated by the elastic properties of titanium implants, cannot be represented by straight-line CT-based planning or navigation systems.

The clinical relevance of these limitations remains insufficiently quantified. Specifically, it is unclear what proportion of screws successfully inserted under fluoroscopic guidance would have been classified as infeasible using standard CT-based rectilinear analysis, and whether postoperative three-dimensional reconstructions demonstrate screw trajectories that depart from idealized straight paths assumed during planning.

The primary objective of this study was therefore to determine the proportion of fluoroscopically inserted pubic ramus screws that could not have been predicted as feasible using conventional CT-based rectilinear planning criteria based on outer thread diameter. Secondary objectives were to assess interobserver reliability for feasibility assessment, to evaluate the impact of core-diameter–based planning on feasibility classification, and to analyze postoperative three-dimensional screw trajectories to determine whether they reflect non-rectilinear pathways incompatible with current CT-based planning and navigation paradigms. We hypothesized that a substantial proportion of accurately positioned pubic ramus screws would not be predicted by standard rectilinear CT planning, highlighting intrinsic limitations of current CT-based planning strategies in anterior pelvic ring fixation.

## Methods

### Study design and patient selection

A retrospective cohort study was conducted at a Level I trauma center. All consecutive patients who underwent fluoroscopy-guided percutaneous screw fixation of superior pubic ramus fractures between January 2022 and December 2025 were screened for inclusion. Institutional review board approval was obtained. The requirement for informed consent was waived due to the retrospective observational design of the study. Patients were eligible if both a complete preoperative pelvic CT scan and a postoperative CT scan were available, allowing assessment of preoperative feasibility and postoperative screw positioning. Patients with incomplete imaging datasets were excluded.

### Preoperative CT-based planning

**Fig. 1:**
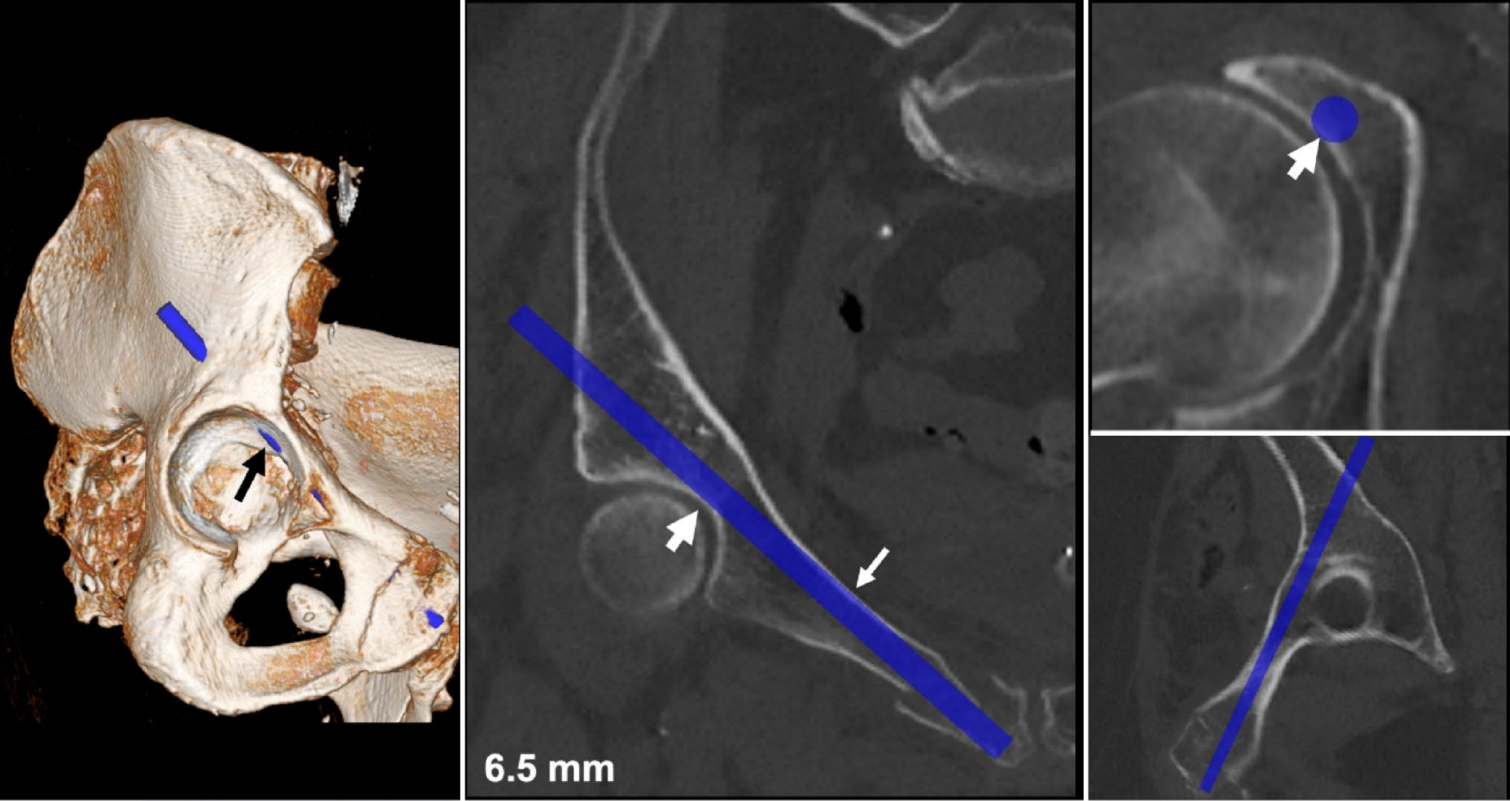
Example of CT-based rectilinear planning using a 6.5 mm cylindrical model. No safe trajectory could be identified, with simulated cortical breach along the medial aspect of the superior pubic ramus (white arrows) and intra-articular penetration of the acetabulum, as demonstrated on three-dimensional reconstruction (black arrow)

For each included patient, Fracture location was classified according to the Nakatani classification based on preoperative CT imaging, allowing differentiation between parasymphyseal, intermediate, and periacetabular regions [8]. Preoperative CT scans were independently reviewed by two independent fellowship-trained pelvic and acetabular surgeons. Observers were blinded to intraoperative findings and postoperative imaging. Preoperative CT-based planning and postoperative three-dimensional reconstructions were performed using dedicated imaging software (Synapse 3D, Fujifilm, Tokyo, Japan). Multiplanar reconstructions (axial, sagittal, and coronal) were systematically used for analysis, with three-dimensional visualization as an adjunct for spatial orientation. CT-based feasibility assessment was performed according to the rectilinear planning methodology described in previously published CT simulation studies [9]. Briefly, a straight cylindrical model (6.5 mm and 7.3 mm diameter), corresponding to the intended screw outer thread diameter, was manually positioned along the intramedullary axis of the superior pubic ramus. This cylindrical simulation, enabled by dedicated 3D planning software, was used to assess the feasibility of a fully intraosseous trajectory. The cylindrical model was first positioned on three-dimensional reconstructions to reproduce the global trajectory of the osseous corridor, and its final position was then meticulously checked on sequential multiplanar reconstructions along the entire ramus; if cortical or intra-articular violation was identified, the cylinder was repositioned and reassessed. Feasibility was defined as the ability to position the cylinder entirely within cancellous bone, without cortical breach or intra-articular penetration along its full length. Each corridor was classified as either feasible or infeasible. Interobserver agreement was subsequently assessed (Fig. 1).

### Surgical technique and postoperative assessment

All procedures were performed using fluoroscopy-guided percutaneous techniques according to standard institutional practice. Procedures were performed in the supine position under fluoroscopic guidance using standard inlet, outlet, and outlet–obturator oblique views, as described in the literature [13]. Screw insertion was performed in either an antegrade or retrograde fashion, with progressive guidewire advancement under continuous fluoroscopic control. Postoperative CT scans were reviewed to assess final screw positioning. Screws were evaluated for cortical breach, intra-articular penetration, or neurovascular compromise. Screws meeting none of these criteria were considered accurately positioned.

### Analysis of discrepant cases

Cases in which screws were classified as infeasible on preoperative rectilinear CT planning but demonstrated accurate postoperative positioning were further analyzed to explore potential explanations for this discrepancy.

**Fig. 2:**
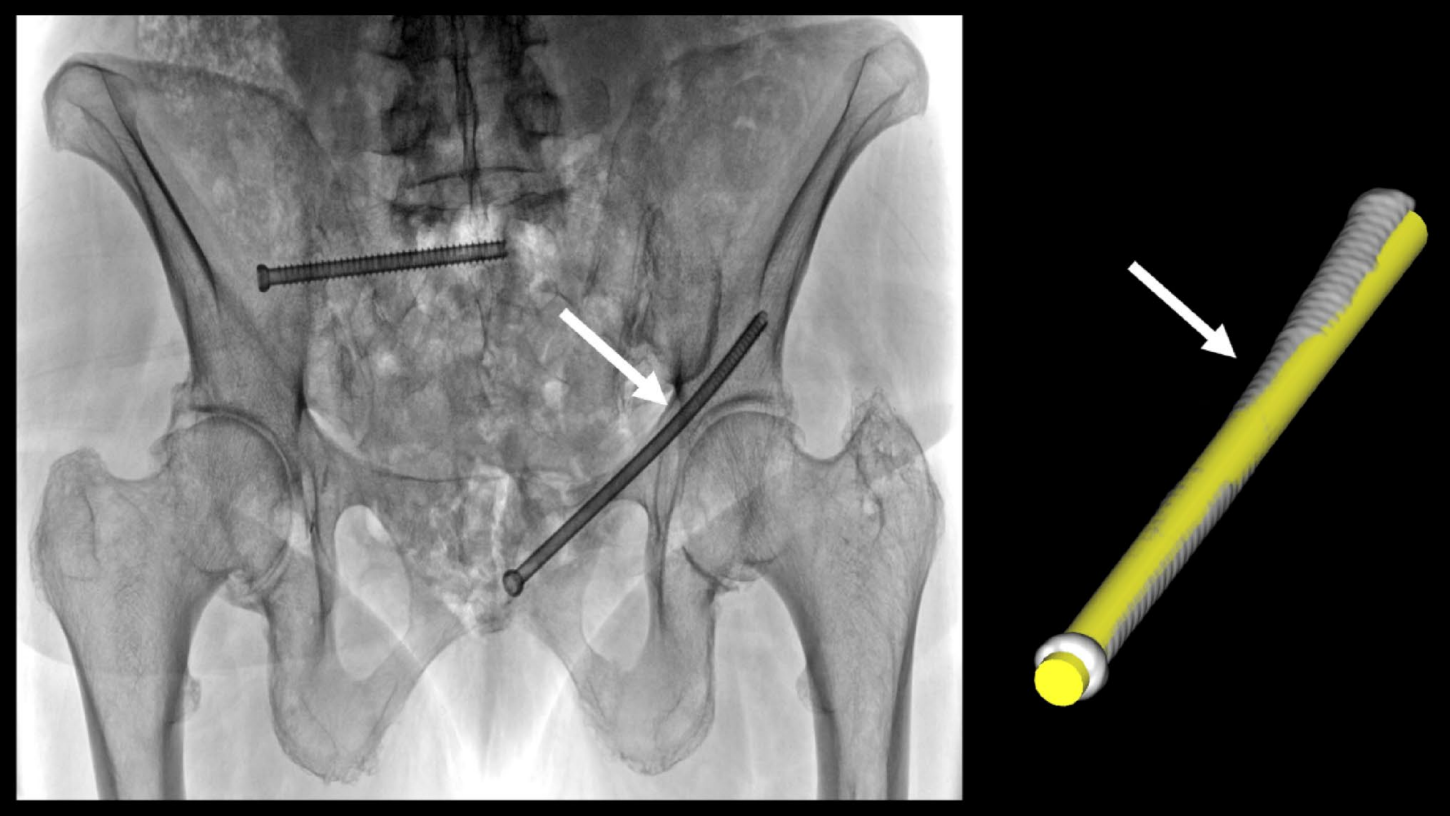
Anteroposterior fluoroscopic view showing a retrograde pubic ramus screw with progressive curvature after crossing the acetabular region. On the right, postoperative three-dimensional CT reconstruction of the same screw with superimposition of a straight 6.5 mm cylindrical model. A marked divergence between the implanted screw and the rectilinear model is observed distal to the acetabulum (white arrow), illustrating guidewire-driven trajectory adaptation within the intramedullary corridor

First, postoperative three-dimensional reconstructions were used to compare the actual screw trajectory with a straight cylindrical model corresponding to the same outer thread diameter. This analysis aimed to assess whether the implanted screw deviated from a rectilinear path, suggesting progressive adaptation of the trajectory during fluoroscopic insertion (Fig. 2).

**Fig. 3:**
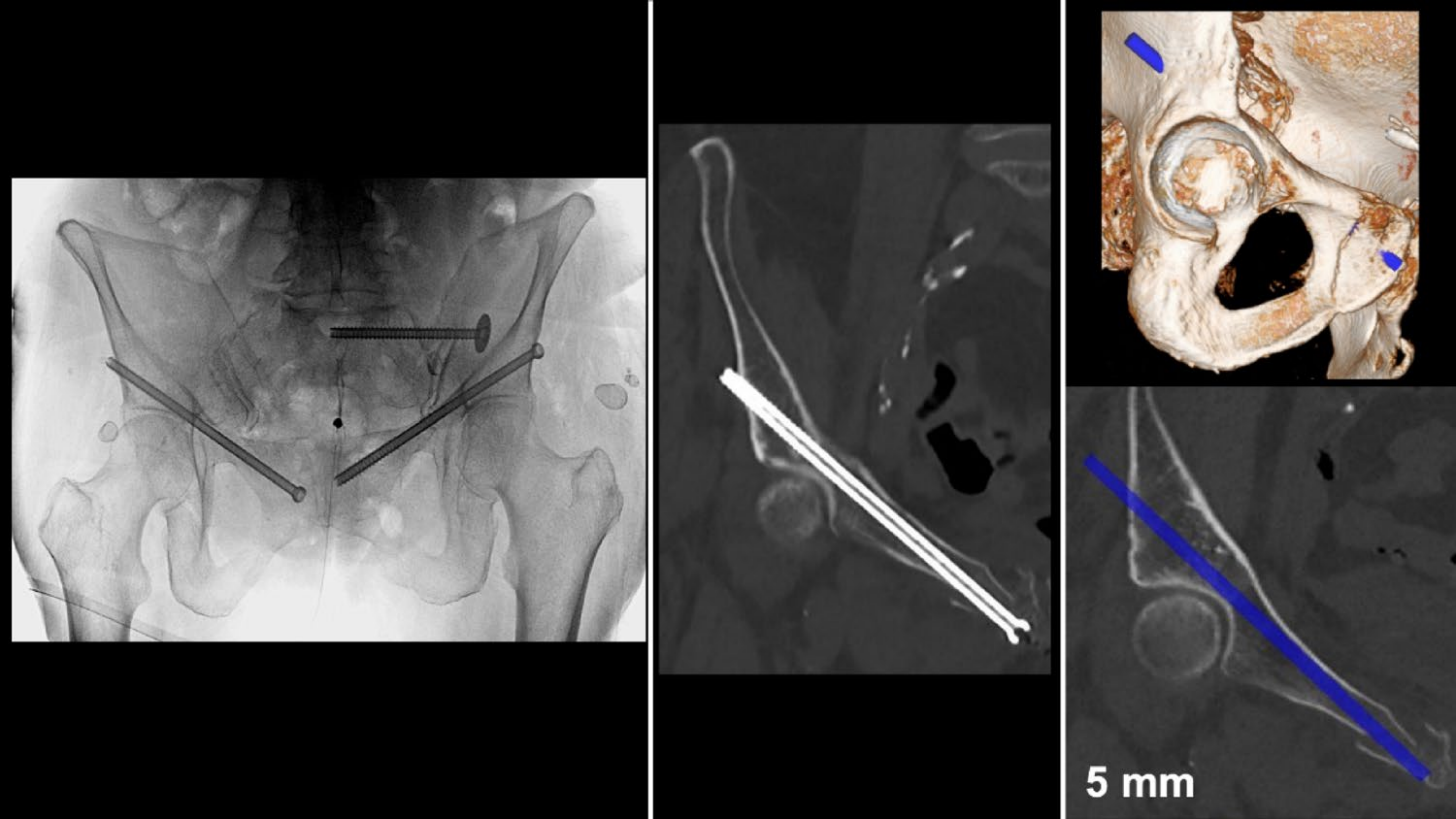
Same patient as in Fig. 1. On the left, anteroposterior fluoroscopic view demonstrating bilateral superior pubic ramus screws accurately positioned. In the center, postoperative CT scan showing the screw located within the intramedullary corridor at a safe distance from the acetabular joint, without cortical breach. On the right, CT-based planning using a 5-mm cylindrical model corresponding to the screw core diameter, demonstrating a feasible intramedullary corridor despite the absence of a feasible trajectory using a 6.5-mm rectilinear model

Second, preoperative CT planning was repeated using a straight cylindrical model corresponding to the core diameter of the implanted screw (5 mm) rather than the outer thread diameter (Fig. 3). Feasibility classification using this alternative criterion was recorded and compared with the original planning results to determine whether core-diameter–based planning improved concordance with postoperative findings.

### Outcome measures

The primary outcome was the proportion of pubic ramus screws that would have been classified as infeasible based on standard CT-based rectilinear planning using outer thread diameter criteria. Secondary outcomes included interobserver reliability for corridor feasibility assessment, postoperative screw positioning accuracy, and the effect of core-diameter–based planning on feasibility classification.

### Statistical analysis

Given the descriptive nature of the study, analyses were primarily descriptive. Categorical variables were reported as frequencies and percentages. Interobserver agreement for CT-based feasibility assessment was evaluated using Cohen’s kappa coefficient. Statistical analyses were performed using using R software, version 3.6.1 (R Foundation for Statistical Computing).

A flowchart summarizing patient inclusion, screw analysis, and outcome assessment.

## Results

The study population included 33 patients, with a slight female predominance (18 women and 15 men). The mean age was 62 ± 18 years (range, 20–91) and BMI 25.8 ± 4.6 kg/m^2^ (18–35). The overall population was characterized by a wide age distribution, reflecting both high-energy trauma in younger patients and fragility fractures in the elderly. According to the Nakatani classification, fracture distribution was as follows: 8 fractures (24%) in zone I, 18 fractures (55%) in zone II, and 7 fractures (21%) in zone III. Patient demographics and fracture characteristics are summarized in Table 1.

**Table 1:** Patient demographics and fracture characteristics

Variable	Value
Patients, n	33
Screws, n	42
Age, mean ± SD (range)	59 ± 20 years (20–91)
Sex	18 women / 15 men
BMI, mean ± SD	25.8 ± 4.6 kg/m^2^ (18–35)
Nakatani classification	
Zone 1	8 (24%)
Zone II	18 (55%)
Zone III	7 (21%)

Patient demographics and fracture characteristics. Values are presented as mean ± standard deviation (range) or number (%). Fracture location was classified according to the Nakatani classification based on preoperative CT imaging.

42 fluoroscopy-guided percutaneous pubic ramus screws were included, including both 6.5-mm and 7.3-mm screws. Using standard CT-based rectilinear planning based on the outer thread diameter corresponding to the implanted screw, 27 screws (64%) were classified as feasible, whereas 15 screws (36%) were deemed infeasible by consensus between the two observers. Interobserver agreement was high (κ = 0.89 [95% CI, 0.75–1.00] for the 6.5 mm model and κ = 0.81 [95% CI, 0.64–0.97] for the 7.3-mm model), with an overall agreement of 90% **(**Table 2**).**

**Table 2:** CT-based planning feasibility and postoperative outcomes for pubic ramus screws

Variable	Value
Screws, n	42
Screw diameter	6.5 mm and 7.3 mm
Feasible (CT rectilinear planning)	27 (64%)
Not feasible (CT rectilinear planning)	15 (36%)
Interobserver agreement	κ = 0.89 [95% CI, 0.75–1.00] (6.5 mm) κ = 0.81 [95% CI, 0.64–0.97] (7.3 mm)
Accurate postoperative positioning	42 (100%)
Cortical breach	0
Intra-articular penetration	0
Neurovascular complication	0
Fixation-related revision surgery	0

CT-based feasibility was assessed using rectilinear planning models corresponding to the outer thread diameter of the implanted screw (6.5 or 7.3 mm). Accurate positioning was defined as the absence of cortical breach, intra-articular penetration, or neurovascular complication on postoperative CT imaging

Postoperative CT imaging demonstrated accurate screw positioning in all cases (100%), with no cortical breach, intra-articular penetration, neurovascular complication, or fixation-related revision surgery. All screws classified as infeasible on preoperative CT planning were successfully inserted and correctly positioned postoperatively **(**Table 3**).**

**Table 3:** Effect of core-diameter–based planning in screws classified as infeasible on standard CT planning

Variable	Value
Screws classified as infeasible (thread diameter)	15
Core diameter used for reassessment	5 mm
Reclassified as feasible with core planning	14 (93%)
Remaining infeasible with core planning	1 (7%)
Postoperative positioning accuracy in this subgroup	100%

Among screws initially classified as infeasible using standard CT-based rectilinear planning based on outer thread diameter, feasibility was reassessed using a straight cylindrical model corresponding to the screw core diameter (5 mm). Fourteen of fifteen screws were reclassified as feasible. One screw remained infeasible despite accurate postoperative intramedullary positioning, reflecting a markedly non-rectilinear trajectory

**Fig. 4:**
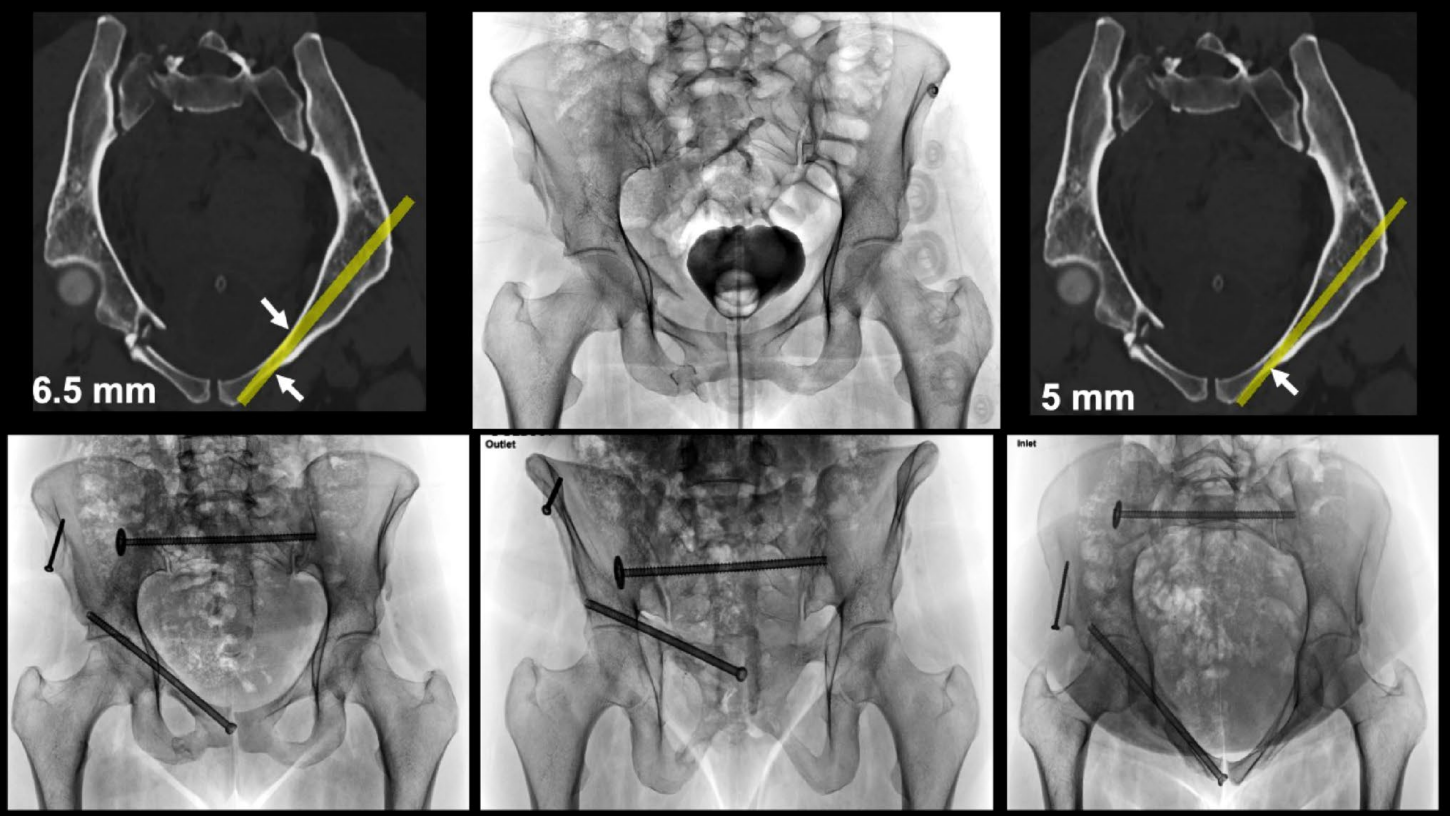
Representative LC-1 pelvic injury, illustrating the unique case in which no rectilinear corridor could be identified even using a core-diameter model Top left: CT-based rectilinear planning on the uninjured left side using a 6.5 mm cylindrical model, demonstrating absence of a safe intramedullary corridor. Top center: Preoperative anteroposterior pelvic radiograph. Top right: CT-based planning on the uninjured side using a 5 mm cylindrical model corresponding to the screw core diameter, again demonstrating no feasible rectilinear corridor. Bottom: Postoperative anteroposterior (left) and inlet (right) radiographs showing accurate reduction and stable fixation despite the absence of a rectilinear corridor on preoperative planning.

**Fig. 5:**
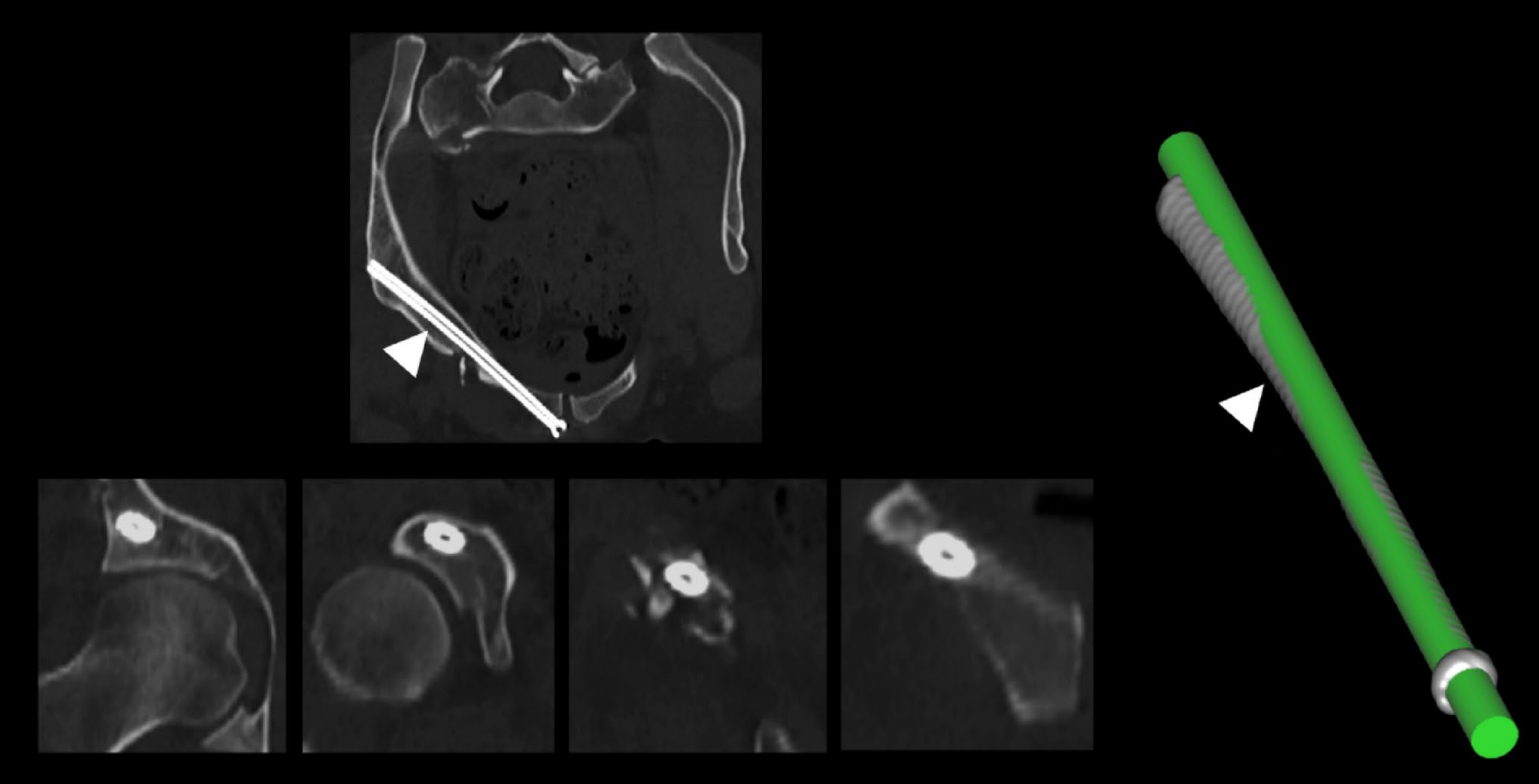
Postoperative CT analysis of the same patient as in Fig. 4, illustrating a markedly non-rectilinear screw trajectory Top left: Axial CT slice through the right superior pubic ramus demonstrating a curved intramedullary screw trajectory. Bottom left: Series of orthogonal CT cross-sections obtained along the axis of the superior pubic ramus from the pubic symphysis to the acetabular roof, showing the screw entirely contained within a narrow intramedullary corridor, at a safe distance from the joint and without cortical breach. Right: Three-dimensional reconstruction of the implanted screw with superimposition of a straight 6.5-mm cylindrical model. A pronounced divergence between the actual screw trajectory and the rectilinear model is observed distal to the symphysis (white arrow), illustrating a degree of trajectory curvature that cannot be approximated by any straight cylindrical planning model.

Among the 15 screws initially classified as infeasible using thread-diameter–based rectilinear planning, all were partially threaded implants. Reassessment using a core-diameter–based (5 mm) cylindrical model reclassified 14 screws (93%) as feasible. One screw, implanted with a 6.5 mm diameter, remained classified as infeasible even with core-diameter–based planning despite accurate postoperative intramedullary positioning. In this case, postoperative three-dimensional reconstruction demonstrated a markedly non-rectilinear screw trajectory that could not be encompassed by any straight cylindrical model, illustrating an extreme example of guidewire-driven trajectory adaptation (Figs. 4 and 5).

## Discussion

The present study demonstrates that standard CT-based rectilinear planning substantially underestimates the feasibility of fluoroscopy-guided pubic ramus screw fixation. More than one third of screws in this cohort would have been classified as infeasible using conventional CT planning criteria based on outer thread diameter, yet all were accurately positioned postoperatively without complication. These findings highlight intrinsic limitations of current planning paradigms and suggest that feasibility is governed by additional geometric and implant-related factors that are not captured by straight-line CT analysis.

A first key finding is the impact of implant geometry on feasibility assessment. Conventional CT planning relies on the outer thread diameter of the screw, implicitly assuming that the entire threaded portion must remain fully intramedullary along the narrowest segment of the corridor. However, between 6.5 mm and 7.3 mm screws, the increase in nominal diameter is primarily related to the size of the threads rather than the core, which remains constant at approximately 5 mm. From a biomechanical standpoint, the trajectory of the screw is primarily defined by its core diameter, while the threads extend beyond this envelope and may contribute to cortical contact [14].

**Fig. 6:**
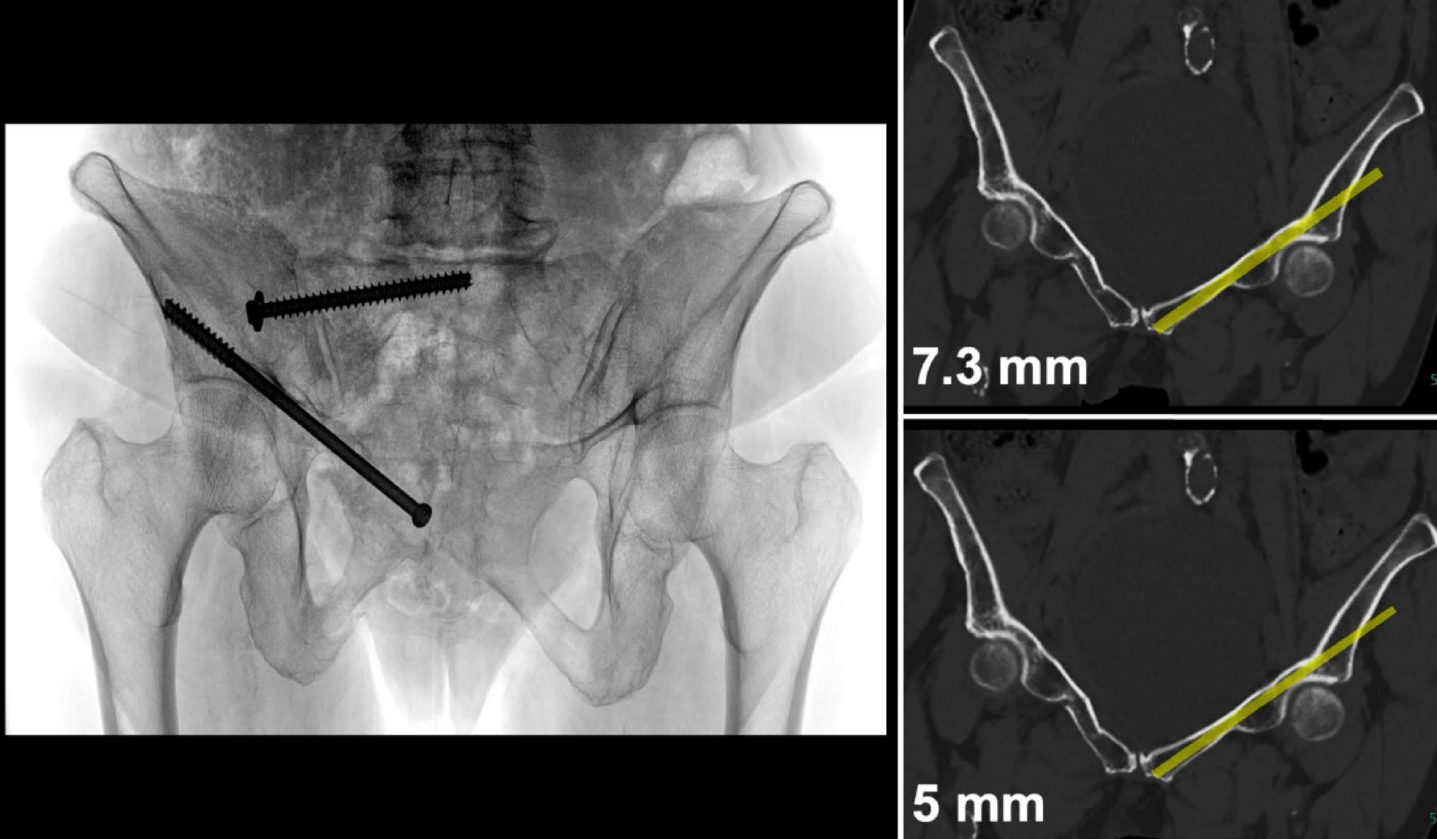
Illustration of partial-threaded screw strategy in a fluoroscopy-guided retrograde pubic ramus fixation. Left: Anteroposterior radiograph showing a retrograde 7.3 mm partially threaded screw, with the threaded portion positioned in the acetabular roof, well away from the joint. Top right: CT-based rectilinear planning using a 7.3 mm cylindrical model demonstrating the absence of a feasible intramedullary corridor, with simulated cortical breach Bottom right: CT-based planning using a 5 mm cylindrical model corresponding to the screw core diameter, demonstrating a feasible intramedullary corridor without cortical violation or false passage. This example illustrates that partial threading allows the narrowest and highest-risk segment of the corridor to be traversed by the screw core alone, while the larger-diameter threaded portion is positioned in a wider, cancellous and safer region of the ramus.

In corridors that are narrow or closely adjacent to the acetabular joint, especially near the most constrained portions of the superior pubic ramus, planning based on thread diameter may therefore be overly restrictive. In such situations, the use of partially threaded screws allows the narrowest segment of the corridor to be traversed by the core alone, while the threaded portion is positioned in wider cancellous regions. This consideration is particularly relevant for 7.3 mm screws, for which thread diameter disproportionately increases relative to the core. In the present study, reassessment using a core-diameter–based planning model improved concordance between preoperative feasibility assessment and postoperative outcomes in the vast majority of cases, supporting the concept that planning based on the screw core better reflects clinical reality. However, planning based on core diameter alone may underestimate the risk of cortical breach during screw insertion, as thread crests extend beyond the core envelope and may transiently violate cortical boundaries, particularly in narrow corridors. Based on these observations, our current practice has shifted toward preferential use of partially threaded screws, except in cases where preoperative planning demonstrates a sufficiently wide intramedullary corridor. In both antegrade and retrograde fixation, this strategy allows the narrowest and highest-risk segment of the corridor, adjacent to the joint and neurovascular structures, to be traversed by the 5 mm core alone (Fig. 6).

A second major mechanism underlying the discrepancy between CT planning and intraoperative feasibility relates to screw trajectory geometry. Standard CT-based planning assumes a rigid, rectilinear trajectory, whereas fluoroscopy-guided insertion relies on a flexible guidewire that can progressively adapt its path within the intramedullary canal [15]. As demonstrated by postoperative three-dimensional reconstructions in this study, several screws followed non-rectilinear trajectories that could not be fully encompassed by a straight cylindrical model corresponding to the outer thread diameter. Notably, in one case, even core-diameter–based rectilinear planning failed to predict feasibility, despite accurate intramedullary screw placement. In this instance, the implanted 6.5 mm screw demonstrated a markedly curved trajectory on postoperative three-dimensional reconstruction, reflecting substantial guidewire deformation within the corridor (Figs. 4 and 5). This extreme example illustrates that feasibility may occasionally depend on trajectories that cannot be approximated by any straight cylindrical model, regardless of diameter, further emphasizing the intrinsic limitations of rectilinear planning assumptions. These findings support the limitations of static geometric planning and suggest that patient-specific biomechanical models simulating fracture displacement may improve planning accuracy [16].

This phenomenon likely reflects guidewire behavior at the bone–implant interface. When encountering areas of denser cortical bone, the guidewire may undergo controlled elastic deformation, gradually redirecting its trajectory toward available cancellous pathways [17]. The definitive screw subsequently follows the established guidewire path. This effect is expected to be more pronounced with titanium screws compared with stainless steel implants, given their greater elasticity. The superposition of curved implanted screw trajectories and rectilinear cylindrical models in this study visually illustrates this limitation of straight-line planning and supports the concept of non-rectilinear “functional corridors” during fluoroscopy-guided fixation. These observations also support the concept of implants designed to better accommodate curved intramedullary pathways. By allowing controlled implant curvature along a guidewire-defined trajectory, such devices may better adapt to the complex anatomy of the pubic ramus and expand the indications for percutaneous fixation in anatomically constrained corridors.

These findings have important implications for the development and clinical use of navigation systems in anterior pelvic ring fixation. Current navigation platforms are fundamentally based on rectilinear trajectories defined on intraoperative CT scans [18] While such systems may accurately identify optimal entry points and reduce variability in starting position for anterior column screw, they are inherently unable to account for curved or progressively adjusted trajectories enabled by guidewire flexibility. As a result, navigation may falsely classify certain corridors as infeasible, potentially discouraging percutaneous fixation in cases that are technically achievable under fluoroscopic guidance.

Rather than negating the value of navigation, the present results suggest that future planning and navigation tools should integrate more flexible geometric models, incorporating core-diameter–based constraints and allowing for non-rectilinear trajectories. Such developments could improve feasibility prediction while preserving the advantages of navigation for entry-point selection and spatial orientation.

This study has several limitations. Its retrospective design may introduce selection bias, and the analysis was performed at a single Level I trauma center with surgeons experienced in fluoroscopy-guided pelvic fixation. The absence of complications limits comparative statistical analysis but reflects the safety of the technique in this cohort. Finally, non-rectilinear trajectories were assessed qualitatively rather than quantified by curvature metrics, although three-dimensional reconstructions clearly demonstrated deviations from straight-line planning assumptions.

## Conclusions

Standard CT-based rectilinear planning underestimates the feasibility of fluoroscopy-guided pubic ramus screw fixation. Planning based on outer thread diameter and rigid trajectories fails to account for implant geometry, partial threading strategies, and guidewire-driven non-rectilinear pathways. Core-diameter–based planning and consideration of functional screw trajectories may better reflect intraoperative realities and should be integrated into future planning and navigation systems for anterior pelvic ring fixation.

## Data Availability

No datasets were generated or analysed during the current study.
